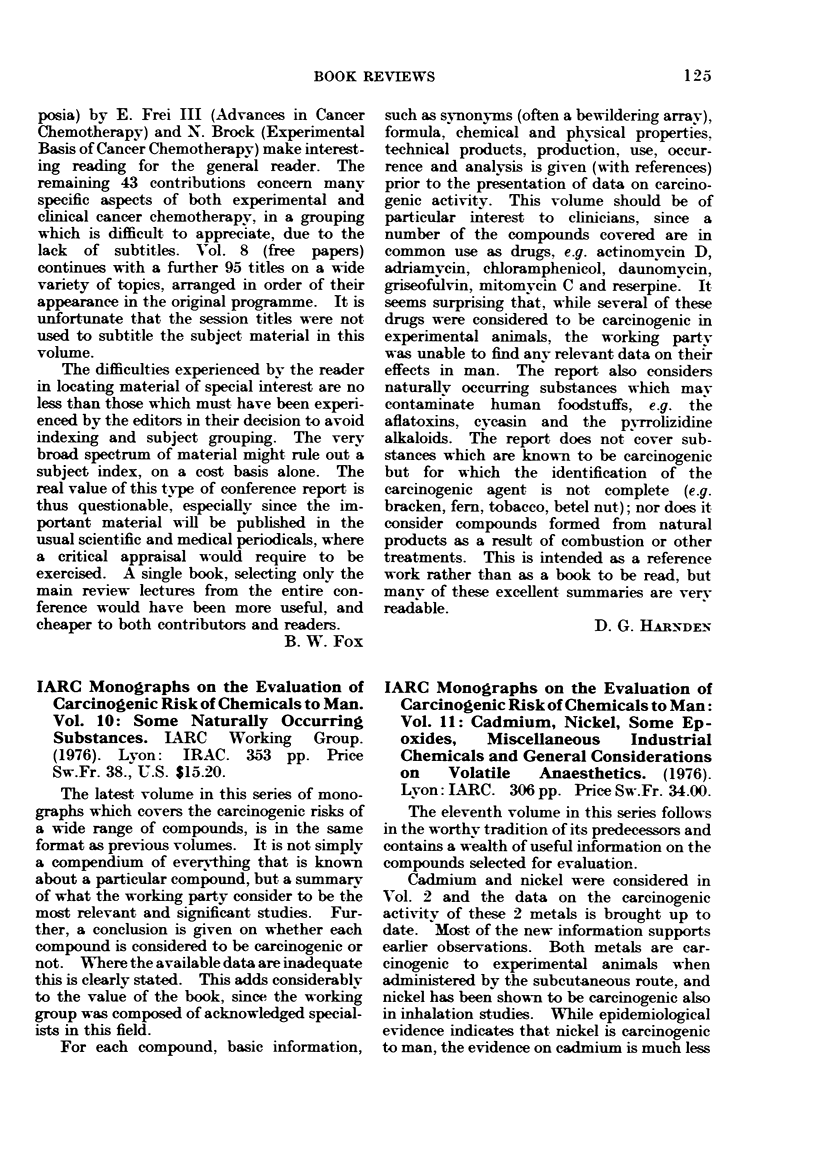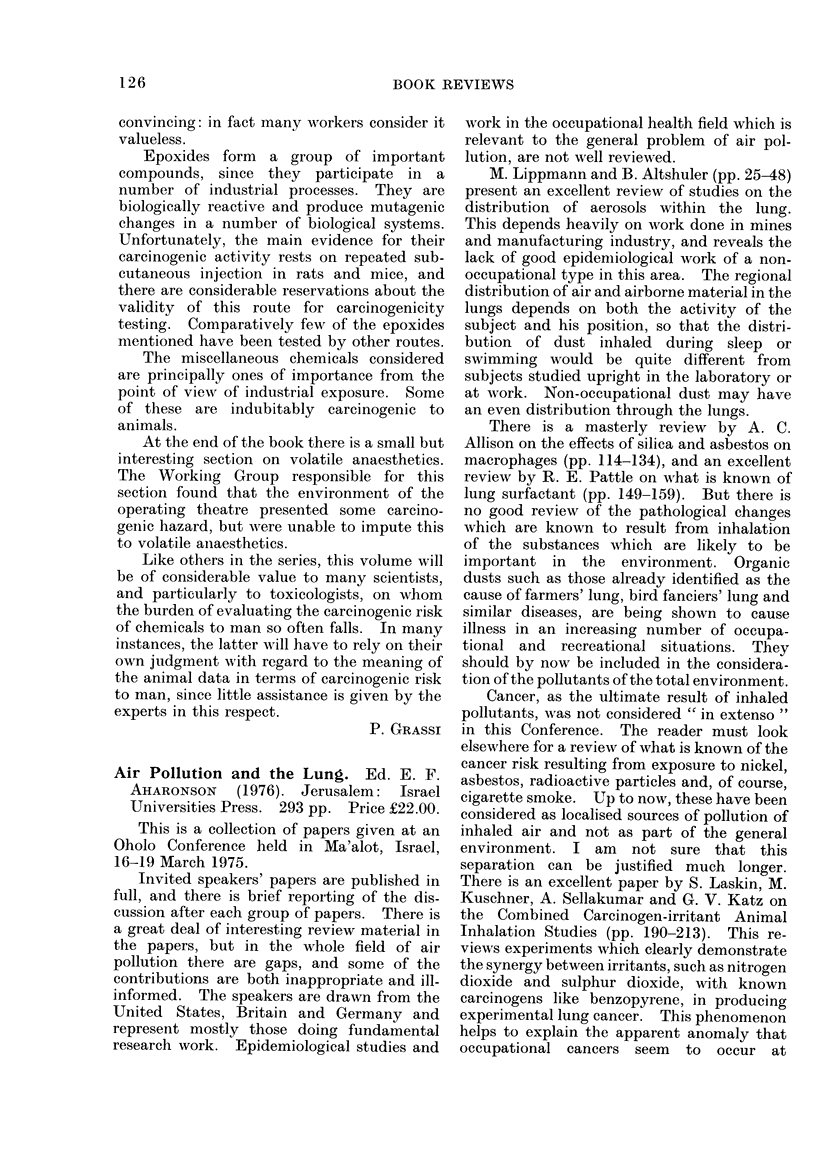# IARC Monographs on the Evaluation of Carcinogenic Risk of Chemicals to Man: Vol. 11: Cadmium, Nickel, Some Epoxides, Miscellaneous Industrial Chemicals and General Considerations on Volatile Anaesthetics

**Published:** 1977-01

**Authors:** P. Grassi


**IARC Monographs on the Evaluation of Carcinogenic Risk of Chemicals to Man: Vol. 11: Cadmium, Nickel, Some Epoxides, Miscellaneous Industrial Chemicals and General Considerations on Volatile Anaesthetics.** (1976). Lyon: IARC. 306 pp. Price Sw.Fr. 34.00.

The eleventh volume in this series follows in the worthy tradition of its predecessors and contains a wealth of useful information on the compounds selected for evaluation.

Cadmium and nickel were considered in Vol. 2 and the data on the carcinogenic activity of these 2 metals is brought up to date. Most of the new information supports earlier observations. Both metals are carcinogenic to experimental animals when administered by the subcutaneous route, and nickel has been shown to be carcinogenic also in inhalation studies. While epidemiological evidence indicates that nickel is carcinogenic to man, the evidence on cadmium is much less convincing: in fact many workers consider it valueless.

Epoxides form a group of important compounds, since they participate in a number of industrial processes. They are biologically reactive and produce mutagenic changes in a number of biological systems. Unfortunately, the main evidence for their carcinogenic activity rests on repeated subcutaneous injection in rats and mice, and there are considerable reservations about the validity of this route for carcinogenicity testing. Comparatively few of the epoxides mentioned have been tested by other routes.

The miscellaneous chemicals considered are principally ones of importance from the point of view of industrial exposure. Some of these are indubitably carcinogenic to animals.

At the end of the book there is a small but interesting section on volatile anaesthetics. The Working Group responsible for this section found that the environment of the operating theatre presented some carcinogenic hazard, but were unable to impute this to volatile anaesthetics.

Like others in the series, this volume will be of considerable value to many scientists, and particularly to toxicologists, on whom the burden of evaluating the carcinogenic risk of chemicals to man so often falls. In many instances, the latter will have to rely on their own judgment with regard to the meaning of the animal data in terms of carcinogenic risk to man, since little assistance is given by the experts in this respect.

P. Grassi